# 2-Bromo-1-mesitylethanone

**DOI:** 10.1107/S1600536809012276

**Published:** 2009-04-22

**Authors:** Lei Chen, Qing-Bing Xu, Guang-Liang Song, Hong-Jun Zhu

**Affiliations:** a Department of Applied Chemistry, College of Science, Nanjing University of Technology, Nanjing 210009, People’s Republic of China

## Abstract

In the mol­ecule of the title compound, C_11_H_13_BrO, the adjacent C atoms are almost coplanar with the aromatic ring [maximum deviation 0.035 (3) Å]. In the crystal structure, weak inter­molecular C—H⋯O inter­actions link the mol­ecules into chains along the *b* axis. A very weak C—H⋯π inter­action is also present.

## Related literature

The title compound is used to synthesize organic electronic devices, see: Rose *et al.* (2008 ▶). For a related structure, see: Guss (1953 ▶). For bond-length data, see: Allen *et al.* (1987 ▶).
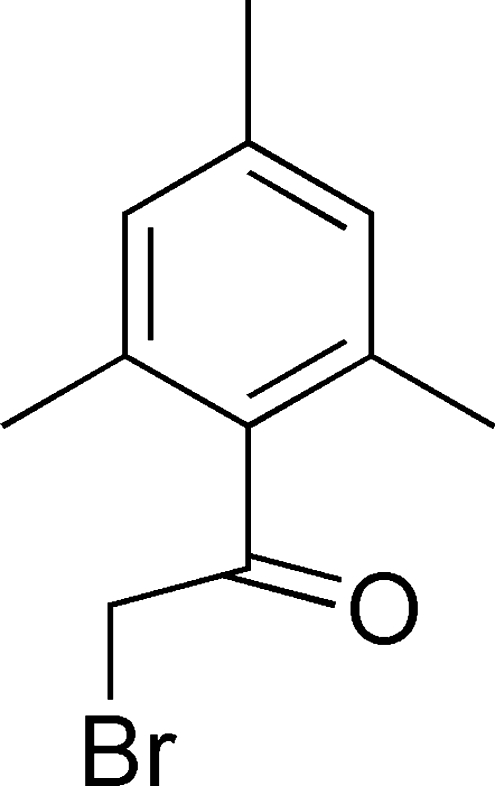


## Experimental

### 

#### Crystal data

C_11_H_13_BrO
*M* *_r_* = 241.12
Orthorhombic, 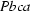

*a* = 15.379 (3) Å
*b* = 8.2820 (17) Å
*c* = 17.374 (4) Å
*V* = 2212.9 (8) Å^3^
*Z* = 8
Mo *K*α radiation
μ = 3.68 mm^−1^
*T* = 294 K
0.20 × 0.10 × 0.10 mm

#### Data collection

Enraf–Nonius CAD-4 diffractometer
Absorption correction: ψ scan (North *et al.*, 1968 ▶) *T*_min_ = 0.527, *T*_max_ = 0.710
3509 measured reflections
2002 independent reflections
805 reflections with *I* > 2σ(*I*)
*R*_int_ = 0.099
3 standard reflections frequency: 120 min intensity decay: 1%

#### Refinement

*R*[*F* ^2^ > 2σ(*F* ^2^)] = 0.064
*wR*(*F* ^2^) = 0.095
*S* = 1.00
2002 reflections
118 parameters
H-atom parameters constrained
Δρ_max_ = 0.28 e Å^−3^
Δρ_min_ = −0.29 e Å^−3^

### 

Data collection: *CAD-4 Software* (Enraf–Nonius, 1985 ▶); cell refinement: *CAD-4 Software*; data reduction: *XCAD4* (Harms & Wocadlo, 1995 ▶); program(s) used to solve structure: *SHELXS97* (Sheldrick, 2008 ▶); program(s) used to refine structure: *SHELXL97* (Sheldrick, 2008 ▶); molecular graphics: *SHELXTL* (Sheldrick, 2008 ▶); software used to prepare material for publication: *SHELXTL*.

## Supplementary Material

Crystal structure: contains datablocks I, global. DOI: 10.1107/S1600536809012276/hk2660sup1.cif

Structure factors: contains datablocks I. DOI: 10.1107/S1600536809012276/hk2660Isup2.hkl

Additional supplementary materials:  crystallographic information; 3D view; checkCIF report

## Figures and Tables

**Table 1:** Hydrogen-bond geometry (Å, °)

*D*—H⋯*A*	*D*—H	H⋯*A*	*D*⋯*A*	*D*—H⋯*A*
C9—H9*B*⋯O^i^	0.96	2.52	3.462 (7)	166
C11—H11*A*⋯O^i^	0.97	2.36	3.308 (7)	167
C7—H7*C*⋯*Cg*1^ii^	0.96	2.94	3.722 (3)	140

Symmetry codes: (i) 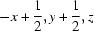; (ii) 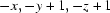. *Cg*1 is the centroid of the C1–C6 ring.

**Fig. 1.**
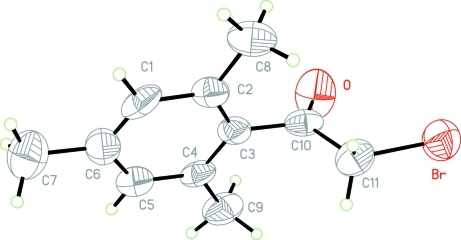
The molecular structure of the title molecule, with the atom-numbering scheme. Displacement ellipsoids are drawn at the 50% probability level.

## supplementary crystallographic information

### Comment

The title compound is used to synthesize organic electronic devices and medical intermediates (Rose *et al.*,  2008). We report herein the crystal structure of the title compound, which is interested to us in the field.

In the molecule of the title compound (Fig. 1), the bond lengths (Allen *et al.*,  1987) and angles are within normal ranges. Ring A (C1-C6) is, of course, planar. Atoms C7, C8, C9 and C10 are -0.012 (2), -0.019 (3), -0.035 (3) and -0.006 (3) Å away from the ring plane of A, respectively.

In the crystal structure, weak intermolecular C-H···O interactions (Table 1) link the molecules into chains along the b axis, in which they may be effective in the stabilization of the structure. There also exists a weak C—H···π interaction (Table 1).

### Experimental

The title compound, (m.p. 323-324 K), was prepared according to the literature method (Guss, 1953). Crystals suitable for X-ray analysis were obtained by dissolving the title compound (0.2 g) in ethyl acetate (50 ml) and evaporating the solvent slowly at room temperature for about 3 d.

### Refinement

H atoms were positioned geometrically, with C-H = 0.93, 0.97 and 0.96 Å for aromatic, methylene and methyl H, respectively, and constrained to ride on their parent atoms, with U_iso_(H) = xU_eq_(C), where x = 1.5 for methyl H and x = 1.2 for all other H atoms.

### Crystal data

**Table:**

C_11_H_13_BrO	*F*(000) = 976
*M**_r_* = 241.12	*D*_x_ = 1.447 Mg m^−^^3^
Orthorhombic, *P**b**c**a*	Mo *K*α radiation, λ = 0.71073 Å
Hall symbol: -P 2ac 2ab	Cell parameters from 25 reflections
*a* = 15.379 (3) Å	θ = 10–13°
*b* = 8.2820 (17) Å	µ = 3.68 mm^−^^1^
*c* = 17.374 (4) Å	*T* = 294 K
*V* = 2212.9 (8) Å^3^	Needle, colorless
*Z* = 8	0.20 × 0.10 × 0.10 mm

### Data collection

**Table:**

Enraf–Nonius CAD-4 diffractometer	805 reflections with *I* > 2σ(*I*)
Radiation source: fine-focus sealed tube	*R*_int_ = 0.099
graphite	θ_max_ = 25.3°, θ_min_ = 2.3°
ω/2θ scans	*h* = 0→18
Absorption correction: ψ scan (North *et al.*, 1968)	*k* = 0→9
*T*_min_ = 0.527, *T*_max_ = 0.710	*l* = −20→12
3509 measured reflections	3 standard reflections every 120 min
2002 independent reflections	intensity decay: 1%

### Refinement

**Table:**

Refinement on *F*^2^	Primary atom site location: structure-invariant direct methods
Least-squares matrix: full	Secondary atom site location: difference Fourier map
*R*[*F*^2^ > 2σ(*F*^2^)] = 0.064	Hydrogen site location: inferred from neighbouring sites
*wR*(*F*^2^) = 0.095	H-atom parameters constrained
*S* = 1.00	*w* = 1/[σ^2^(*F*_o_^2^) + (0.022*P*)^2^] where *P* = (*F*_o_^2^ + 2*F*_c_^2^)/3
2002 reflections	(Δ/σ)_max_ < 0.001
118 parameters	Δρ_max_ = 0.28 e Å^−^^3^
0 restraints	Δρ_min_ = −0.29 e Å^−^^3^

### Special details

**Table:**

Geometry. All e.s.d.'s (except the e.s.d. in the dihedral angle between two l.s. planes) are estimated using the full covariance matrix. The cell e.s.d.'s are taken into account individually in the estimation of e.s.d.'s in distances, angles and torsion angles; correlations between e.s.d.'s in cell parameters are only used when they are defined by crystal symmetry. An approximate (isotropic) treatment of cell e.s.d.'s is used for estimating e.s.d.'s involving l.s. planes.
Refinement. Refinement of *F*^2^ against ALL reflections. The weighted *R*-factor *wR* and goodness of fit *S* are based on *F*^2^, conventional *R*-factors *R* are based on *F*, with *F* set to zero for negative *F*^2^. The threshold expression of *F*^2^ > σ(*F*^2^) is used only for calculating *R*-factors(gt) *etc*. and is not relevant to the choice of reflections for refinement. *R*-factors based on *F*^2^ are statistically about twice as large as those based on *F*, and *R*- factors based on ALL data will be even larger.

### Fractional atomic coordinates and isotropic or equivalent isotropic displacement parameters (Å^2^)

**Table:**

	*x*	*y*	*z*	*U*_iso_*/*U*_eq_	
Br	0.22106 (4)	0.01688 (8)	0.59900 (4)	0.0758 (3)	
O	0.1783 (3)	−0.1396 (5)	0.7510 (3)	0.0853 (16)	
C1	−0.0250 (4)	0.1475 (7)	0.8818 (4)	0.0557 (19)	
H1A	−0.0852	0.1583	0.8816	0.067*	
C2	0.0138 (4)	0.0860 (6)	0.8160 (4)	0.0461 (16)	
C3	0.1038 (4)	0.0675 (6)	0.8208 (4)	0.0458 (17)	
C4	0.1509 (4)	0.1135 (7)	0.8840 (4)	0.0462 (17)	
C5	0.1083 (4)	0.1744 (7)	0.9464 (4)	0.0540 (18)	
H5A	0.1400	0.2040	0.9898	0.065*	
C6	0.0180 (5)	0.1932 (7)	0.9465 (4)	0.062 (2)	
C7	−0.0290 (3)	0.2598 (7)	1.0151 (4)	0.079 (2)	
H7A	−0.0903	0.2624	1.0048	0.119*	
H7B	−0.0088	0.3672	1.0255	0.119*	
H7C	−0.0181	0.1924	1.0590	0.119*	
C8	−0.0388 (3)	0.0364 (6)	0.7488 (3)	0.0630 (18)	
H8A	−0.0989	0.0601	0.7583	0.094*	
H8B	−0.0319	−0.0774	0.7403	0.094*	
H8C	−0.0196	0.0944	0.7040	0.094*	
C9	0.2492 (3)	0.0917 (7)	0.8895 (3)	0.068 (2)	
H9A	0.2692	0.1304	0.9385	0.101*	
H9B	0.2769	0.1517	0.8491	0.101*	
H9C	0.2633	−0.0207	0.8843	0.101*	
C10	0.1514 (4)	−0.0002 (8)	0.7513 (3)	0.0459 (15)	
C11	0.1681 (3)	0.1137 (7)	0.6882 (3)	0.0592 (19)	
H11A	0.2057	0.1991	0.7069	0.071*	
H11B	0.1135	0.1628	0.6729	0.071*	

### Atomic displacement parameters (Å^2^)

**Table:**

	*U*^11^	*U*^22^	*U*^33^	*U*^12^	*U*^13^	*U*^23^
Br	0.0835 (6)	0.0705 (5)	0.0734 (5)	−0.0009 (5)	0.0146 (4)	−0.0066 (5)
O	0.111 (4)	0.057 (3)	0.088 (4)	0.040 (3)	0.016 (3)	0.015 (3)
C1	0.033 (4)	0.044 (4)	0.090 (6)	0.000 (3)	0.021 (4)	0.011 (4)
C2	0.037 (5)	0.036 (4)	0.065 (5)	0.002 (3)	0.000 (4)	−0.005 (4)
C3	0.044 (5)	0.030 (4)	0.064 (5)	−0.002 (3)	0.011 (4)	−0.009 (3)
C4	0.026 (4)	0.041 (4)	0.072 (6)	−0.004 (3)	0.005 (4)	0.001 (4)
C5	0.047 (5)	0.050 (4)	0.065 (5)	−0.008 (4)	−0.004 (4)	0.002 (4)
C6	0.087 (7)	0.039 (4)	0.059 (6)	−0.005 (5)	0.016 (5)	0.002 (4)
C7	0.075 (6)	0.084 (5)	0.079 (7)	0.015 (4)	0.011 (5)	0.009 (5)
C8	0.049 (4)	0.054 (4)	0.085 (5)	0.000 (4)	−0.021 (4)	0.002 (4)
C9	0.048 (5)	0.080 (5)	0.075 (5)	−0.016 (3)	−0.015 (4)	0.014 (4)
C10	0.038 (4)	0.038 (4)	0.061 (4)	−0.005 (4)	−0.016 (3)	0.010 (5)
C11	0.042 (4)	0.065 (5)	0.070 (5)	0.002 (3)	0.003 (4)	0.003 (4)

### Geometric parameters (Å, °)

**Table:**

Br—C11	1.925 (6)	C6—C7	1.499 (8)
O—C10	1.226 (6)	C7—H7A	0.9600
C1—C6	1.358 (9)	C7—H7B	0.9600
C1—C2	1.387 (8)	C7—H7C	0.9600
C1—H1A	0.9300	C8—H8A	0.9600
C2—C3	1.396 (7)	C8—H8B	0.9600
C2—C8	1.479 (7)	C8—H8C	0.9600
C3—C4	1.369 (8)	C9—H9A	0.9600
C3—C10	1.519 (8)	C9—H9B	0.9600
C4—C5	1.364 (7)	C9—H9C	0.9600
C4—C9	1.526 (7)	C10—C11	1.470 (7)
C5—C6	1.399 (7)	C11—H11A	0.9700
C5—H5A	0.9300	C11—H11B	0.9700
			
C6—C1—C2	125.2 (6)	H7B—C7—H7C	109.5
C6—C1—H1A	117.4	C2—C8—H8A	109.5
C2—C1—H1A	117.4	C2—C8—H8B	109.5
C1—C2—C3	114.7 (6)	H8A—C8—H8B	109.5
C1—C2—C8	121.2 (6)	C2—C8—H8C	109.5
C3—C2—C8	124.0 (6)	H8A—C8—H8C	109.5
C4—C3—C2	122.8 (6)	H8B—C8—H8C	109.5
C4—C3—C10	119.0 (6)	C4—C9—H9A	109.5
C2—C3—C10	118.1 (6)	C4—C9—H9B	109.5
C5—C4—C3	119.1 (6)	H9A—C9—H9B	109.5
C5—C4—C9	118.0 (6)	C4—C9—H9C	109.5
C3—C4—C9	122.8 (6)	H9A—C9—H9C	109.5
C4—C5—C6	121.3 (6)	H9B—C9—H9C	109.5
C4—C5—H5A	119.4	O—C10—C11	122.8 (6)
C6—C5—H5A	119.4	O—C10—C3	120.9 (5)
C1—C6—C5	116.8 (7)	C11—C10—C3	116.1 (6)
C1—C6—C7	121.8 (7)	C10—C11—Br	114.0 (4)
C5—C6—C7	121.4 (8)	C10—C11—H11A	108.7
C6—C7—H7A	109.5	Br—C11—H11A	108.7
C6—C7—H7B	109.5	C10—C11—H11B	108.7
H7A—C7—H7B	109.5	Br—C11—H11B	108.7
C6—C7—H7C	109.5	H11A—C11—H11B	107.6
H7A—C7—H7C	109.5		
			
C6—C1—C2—C3	2.1 (9)	C9—C4—C5—C6	−178.0 (5)
C6—C1—C2—C8	178.8 (6)	C2—C1—C6—C5	−0.8 (10)
C1—C2—C3—C4	−3.0 (8)	C2—C1—C6—C7	179.6 (6)
C8—C2—C3—C4	−179.6 (6)	C4—C5—C6—C1	0.2 (9)
C1—C2—C3—C10	179.6 (5)	C4—C5—C6—C7	179.9 (6)
C8—C2—C3—C10	3.0 (8)	C4—C3—C10—O	77.5 (8)
C2—C3—C4—C5	2.6 (9)	C2—C3—C10—O	−105.0 (7)
C10—C3—C4—C5	180.0 (5)	C4—C3—C10—C11	−98.3 (6)
C2—C3—C4—C9	179.4 (5)	C2—C3—C10—C11	79.2 (7)
C10—C3—C4—C9	−3.3 (8)	O—C10—C11—Br	8.5 (8)
C3—C4—C5—C6	−1.2 (9)	C3—C10—C11—Br	−175.8 (4)

### Hydrogen-bond geometry (Å, °)

**Table:**

*D*—H···*A*	*D*—H	H···*A*	*D*···*A*	*D*—H···*A*
C9—H9B···O^i^	0.96	2.52	3.462 (7)	166
C11—H11A···O^i^	0.97	2.36	3.308 (7)	167
C7—H7C···Cg1^ii^	0.96	2.94	3.722 (3)	140

Symmetry codes: (i) −*x*+1/2, *y*+1/2, *z*; (ii) −*x*, −*y*+1, −*z*+1.

## References

[bb1] Allen, F. H., Kennard, O., Watson, D. G., Brammer, L., Orpen, A. G. & Taylor, R. (1987). *J. Chem. Soc. Perkin Trans. 2*, pp. S1–19.

[bb2] Enraf–Nonius (1985). *CAD-4 Software* Enraf–Nonius, Delft, The Netherlands.

[bb3] Guss, C. O. (1953). *J. Am. Chem. Soc.* **75**, 3177–3179.

[bb4] Harms, K. & Wocadlo, S. (1995). *XCAD4* University of Marburg, Germany.

[bb5] North, A. C. T., Phillips, D. C. & Mathews, F. S. (1968). *Acta Cryst.* A**24**, 351–359.

[bb6] Rose, J. M., Deplace, F., Lynd, N. A., Wang, Z., Hotta, A., Lobkovsky, E. B., Edward, J., Kramer, E. K. & Coates, G. W. (2008). *Macromolecules*, **41**, 9548–9555.

[bb7] Sheldrick, G. M. (2008). *Acta Cryst.* A**64**, 112–122. 10.1107/S0108767307043930 18156677

